# Nicotinamide Supplementation in Glaucoma: A Systematic Review of Human Studies

**DOI:** 10.3390/nu18172885

**Published:** 2026-09-03

**Authors:** Andrea Giudiceandrea, Martina Cocuzza, Gianni Gravina, Valeria Silvestri, Filippo Amore, Marco Sulfaro, Francesco De Dominicis, Simone Bruzio, Epifanio Giudiceandrea, Matteo Salgarello, Grazia Maria Cozzupoli, Maria Cristina Savastano, Benedetto Falsini, Stanislao Rizzo, Tommaso Salgarello

**Affiliations:** 1 Department of Ophthalmology, Fondazione Policlinico Universitario A. Gemelli IRCCS, 00168 Rome, Italy; andrea.giudiceandrea@policlinicogemelli.it (A.G.); simone.bruzio@guest.policlinicogemelli.it (S.B.); mariacristina.savastano@policlinicogemelli.it (M.C.S.); stanislao.rizzo@policlinicogemelli.it (S.R.); tommaso.salgarello@policlinicogemelli.it (T.S.); 2 Faculty of Medicine and Surgery, Università Cattolica del Sacro Cuore, 00168 Rome, Italy; matsalga16@gmail.com (M.S.); bfalsini@gmail.com (B.F.); 3 Department of Ophthalmology, Policlinico San Matteo, 27100 Pavia, Italy; gianni.gravina23@gmail.com; 4 National Centre of Services and Research for the Prevention of Blindness and Rehabilitation of Low Vision Patients-IAPB ETS, Fondazione Policlinico Universitario A. Gemelli IRCCS, 00168 Rome, Italy; v.silvestri@iapb.it (V.S.); f.amore@iapb.it (F.A.); m.sulfaro@iapb.it (M.S.); francesco.dedominicis@guest.policlinicogemelli.it (F.D.D.); 5 Department of Ophthalmology, Azienda Ospedaliera San Paolo, 20142 Milan, Italy; epifanio.giudiceandrea@unimi.it; 6 Department of Ophthalmology, Ospedale dei Castelli, 00040 Ariccia, Italy; mgcozzupoli@gmail.com

**Keywords:** glaucoma, nicotinamide, NAD, neuroprotection, mitochondrial dysfunction, human studies

## Abstract

**Background/Objectives:** Nicotinamide (NAM), a form of vitamin B3 and precursor of nicotinamide adenine dinucleotide (NAD), has emerged as a potential metabolic and neuroprotective strategy in glaucoma. This systematic review aimed to evaluate the available human evidence on NAM-containing and NAD-targeted strategies in glaucoma, integrating interventional, observational, and mechanistic evidence with particular attention to clinical outcomes, methodological quality, and safety. **Methods:** A systematic literature search was conducted in PubMed, Scopus, and the Cochrane Central Register of Controlled Trials (CENTRAL), with the search updated through 22 August 2026. Eligible studies included randomized and non-randomized interventional studies evaluating NAM-containing or NAD-targeted supplementation strategies, as well as observational human studies investigating NAM/NAD-related biomarkers or mitochondrial metabolic alterations in relation to glaucoma. Risk of bias was assessed according to study design using RoB 2 for randomized trials, ROBINS-I for non-randomized interventional studies, and a domain-based assessment for observational exposure and biomarker studies. Owing to substantial clinical and methodological heterogeneity, findings were synthesized narratively without quantitative pooling. **Results:** Thirteen eligible reports representing eight unique human studies were included. Randomized trials provided preliminary evidence that NAM-containing interventions may produce short-term functional and electrophysiological effects. NAM supplementation was associated with improvement in inner retinal electrophysiological function in randomized crossover studies, whereas effects on visual field outcomes were inconsistent. A trial combining NAM with pyruvate reported favorable short-term visual field changes, although the specific contribution of NAM could not be isolated. Non-randomized studies provided complementary evidence on vascular, metabolic, structural, electrophysiological, and patient-reported outcomes but were limited by potential confounding and uncontrolled study designs. Observational studies reported associations between glaucoma and altered circulating NAM concentrations and systemic mitochondrial dysfunction. Available studies were generally small and of limited duration, and the evidence was insufficient to establish sustained neuroprotection or modification of glaucoma progression. **Conclusions:** Human evidence supports the biological plausibility of targeting NAM/NAD metabolism in glaucoma and provides preliminary signals of short-term biological and functional effects. However, current evidence remains insufficient to support routine high-dose NAM supplementation in clinical practice. Larger, adequately powered, long-term randomized controlled trials are required to establish sustained efficacy, optimal dosing, patient selection, and long-term safety.

## 1. Introduction

Glaucoma is a leading cause of irreversible blindness worldwide, with the number of affected individuals projected to reach 111.8 million by 2040 [1]. The disease is characterized by progressive optic neuropathy involving degeneration of retinal ganglion cells (RGCs) and their axons, resulting in structural optic nerve damage and corresponding visual field loss [2].

Elevated intraocular pressure (IOP) remains the most important modifiable risk factor and the primary target of current medical, laser, and surgical treatments [2,3]. However, a substantial proportion of patients continue to experience disease progression despite apparently adequate IOP control, while others with ocular hypertension never develop glaucomatous damage. Conversely, patients with normal-tension glaucoma may show progressive visual loss in the absence of elevated IOP. These observations highlight the contribution of pressure-independent mechanisms to glaucomatous neurodegeneration [2,4].

Glaucoma is increasingly recognized as a multifactorial optic neuropathy in which biomechanical stress, vascular dysregulation, oxidative stress, neuroinflammatory mechanisms, impaired axonal transport, and mitochondrial dysfunction may interact in determining RGC susceptibility [4,5,6]. Among these mechanisms, mitochondrial and metabolic dysfunction have attracted increasing attention because RGCs and their axons have substantial energetic requirements and are particularly vulnerable to impaired cellular energy metabolism [5,6,7].

These considerations have prompted interest in therapeutic strategies that extend beyond IOP lowering and target cellular metabolism, mitochondrial resilience, and neuroprotection. Nicotinamide (NAM), a form of vitamin B3 and a major precursor of nicotinamide adenine dinucleotide (NAD), has emerged as a potential metabolic strategy in glaucoma. NAD is essential for cellular redox reactions, energy metabolism, and multiple signaling pathways, and alterations in NAD homeostasis have been implicated in age-related metabolic vulnerability [8,9,10]. In experimental glaucoma, age-related NAD depletion and mitochondrial dysfunction have been observed before substantial neurodegeneration, while NAM supplementation markedly increased retinal NAD availability and protected RGCs against glaucomatous damage [11]. These findings provide a strong mechanistic rationale for investigating NAD-targeted interventions in patients with glaucoma.

The potential role of NAM and other NAD-related metabolic strategies in glaucoma has been addressed by several previous publications. Bhartiya discussed NAM-mediated neuroprotection as an emerging therapeutic concept in glaucoma [12], while subsequent narrative reviews examined the biological rationale and available preclinical and clinical evidence concerning NAD and vitamin B3 supplementation [13,14,15]. More recently, Sherratt-Mayhew et al. conducted a systematic review specifically evaluating the effect of NAM-containing interventions on visual field mean deviation in randomized clinical trials [16]. Nevertheless, the human literature extends beyond visual field mean deviation and includes electrophysiological outcomes, other visual field parameters, systemic NAD-related metabolic alterations, and observational evidence concerning vitamin B3 status and glaucoma.

The present systematic review was therefore designed to provide an updated and broader appraisal of the available human evidence, distinguishing randomized clinical trials, non-randomized interventional studies, and observational and metabolic evidence and critically evaluating clinical and functional outcomes, methodological quality, safety, and the limitations of the current evidence. This approach aims to clarify the extent to which the strong biological rationale for NAD-targeted interventions is currently supported by human data and to identify the principal evidence gaps that must be addressed before NAM supplementation can be considered for routine glaucoma management.

### 1.1. Mitochondrial Function in Glaucoma

Retinal ganglion cells and their axons have substantial energetic requirements and depend on efficient mitochondrial function to maintain membrane potential, axonal transport, and neuronal signaling [5,6,7]. The unmyelinated portion of RGC axons is particularly metabolically demanding and contains a high density of mitochondria to support continuous energy requirements [5,7].

Mitochondrial dysfunction has increasingly been implicated in glaucomatous neurodegeneration. Experimental evidence indicates alterations in mitochondrial morphology, dynamics, and function, together with impaired cellular capacity to respond to metabolic stress [6,7,17,18]. These abnormalities may compromise ATP production, interfere with axonal transport, and increase RGC susceptibility to additional mechanical, vascular, and metabolic stressors [5,6,7].

Importantly, metabolic and mitochondrial alterations may occur early in the neurodegenerative process. In the DBA/2J mouse model of glaucoma, age-related metabolic vulnerability and NAD depletion were identified before substantial neurodegeneration, suggesting that impaired metabolic resilience may contribute to RGC susceptibility rather than solely representing a consequence of advanced neuronal loss [11]. Age-related alterations in cellular metabolism may therefore reduce the capacity of RGCs to withstand glaucomatous stress.

Mitochondrial dysfunction is also closely associated with oxidative stress. Impaired mitochondrial homeostasis can promote excessive generation of reactive oxygen species and reduce the ability of RGCs to tolerate metabolic challenges, while alterations in mitochondrial dynamics and quality-control mechanisms may facilitate the accumulation of dysfunctional mitochondria [6,7,18]. Together, these processes may contribute to cellular stress and ultimately RGC and axonal degeneration.

Human evidence provides complementary support for mitochondrial involvement in glaucoma. Van Bergen et al. identified alterations in systemic mitochondrial function in patients with advanced primary open-angle glaucoma [19]. In addition, reduced circulating NAM concentrations have been reported in patients with primary open-angle glaucoma [20]. More recently, Petriti et al. demonstrated an association between impaired systemic mitochondrial respiratory function, altered NAD-related metabolism, and faster visual field progression in glaucoma [21]. Although these findings do not establish causality, they support a relationship between metabolic dysfunction and glaucomatous disease progression.

Collectively, experimental and human evidence suggests that mitochondrial dysfunction and impaired metabolic resilience are relevant components of glaucomatous neurodegeneration. This provides a biological rationale for investigating interventions aimed at preserving cellular energy metabolism and NAD homeostasis as potential adjuncts to conventional IOP-lowering therapy.

### 1.2. NAD Metabolism and Vitamin B3 Precursors

Nicotinamide adenine dinucleotide is an essential pyridine nucleotide involved in cellular energy metabolism, redox reactions, DNA repair, gene regulation, and intracellular signaling [8,9,10]. In its oxidized and reduced forms (NAD^+^/NADH), NAD serves as a central redox cofactor linking glycolysis, the tricarboxylic acid cycle, and mitochondrial oxidative phosphorylation. NAD^+^ also acts as a substrate for NAD-consuming enzymes, including sirtuins, poly (ADP-ribose) polymerases (PARPs), CD38, and SARM1, thereby linking cellular NAD availability to metabolic regulation, stress responses, DNA repair, and axonal integrity [9,10,22].

Vitamin B3 occurs principally as nicotinic acid (NA) and nicotinamide (NAM), while nicotinamide riboside (NR) represents an additional NAD precursor. Although NA, NAM, and NR can all contribute to NAD biosynthesis, they enter the metabolic network through different routes [8,9,10]. NA is incorporated through the Preiss–Handler pathway, NAM is predominantly recycled through the NAD salvage pathway, and NR can be phosphorylated by nicotinamide riboside kinases (NRKs) to nicotinamide mononucleotide (NMN), which is subsequently converted to NAD [8,9,10].

These biochemical differences are relevant when considering NAD-targeted supplementation. NAM is both a dietary form of vitamin B3 and a major product of NAD-consuming reactions, allowing it to be continuously recycled through the salvage pathway [8,9,10]. NR can enter NAD biosynthesis through phosphorylation to NMN, although its metabolic fate depends on tissue-specific precursor availability and enzymatic activity. NA also contributes to NAD synthesis but differs pharmacologically from NAM and is characteristically associated with prostaglandin-mediated cutaneous flushing at pharmacological doses [8]. Accordingly, NA, NAM, and NR should not be considered interchangeable when interpreting their biological effects, tolerability, or potential therapeutic applications.

NAD metabolism is closely linked to mitochondrial function because adequate NAD^+^ availability is required to sustain oxidative metabolism and ATP production [9,10]. Perturbations of the NAD^+^/NADH balance may impair mitochondrial respiration and cellular adaptation to energetic stress. In parallel, the NAD phosphate redox couple (NADP^+^/NADPH) contributes to antioxidant defense by supplying reducing equivalents to systems including glutathione and thioredoxin [9,10]. Disruption of NAD homeostasis may therefore affect both cellular bioenergetics and resistance to oxidative stress.

In glaucoma, alterations in NAD-related metabolism have been implicated in RGC vulnerability. Experimental studies have demonstrated age-related reductions in retinal NAD availability and increased susceptibility to metabolic stress, whereas NAM supplementation restored NAD availability and provided substantial RGC protection in glaucoma models [11]. Human studies provide complementary evidence: plasma NAM concentrations have been reported to be lower in patients with primary open-angle glaucoma than in controls [20], while impaired mitochondrial respiratory function and alterations in intracellular NAD metabolism have been associated with faster visual field progression [21].

Together, these findings provide a biological rationale for targeting NAD metabolism in glaucoma. Among available NAD precursors, NAM has received particular attention because of its direct involvement in the salvage pathway and the substantial experimental evidence supporting neuroprotective effects in RGCs [11]. Nevertheless, differences in precursor metabolism, tissue availability, pharmacological effects, and safety profiles must be considered when interpreting studies of NAM, NA, and NR.

### 1.3. NAD Biosynthesis and Metabolism of NAD Precursors

NAD is synthesized and maintained through three principal biosynthetic routes: the salvage pathway, the Preiss–Handler pathway, and de novo synthesis from tryptophan [8,9,10]. These pathways converge on the maintenance of intracellular NAD pools but differ according to the precursor used and the enzymatic machinery involved.

As outlined above, NAM is recycled to NAD^+^ through the salvage pathway via nicotinamide phosphoribosyltransferase (NAMPT) and nicotinamide mononucleotide adenylyltransferases (NMNATs), whereas nicotinic acid enters NAD biosynthesis through the Preiss–Handler pathway and nicotinamide riboside can be converted to NMN through nicotinamide riboside kinases (NRKs) [8,9,10]. NAD may also be synthesized de novo from tryptophan through the kynurenine pathway [9,10].

Importantly, the metabolic fate of orally administered NAD precursors is more complex than these canonical intracellular pathways suggest. Using isotope tracing, Chellappa et al. showed that dietary NAD precursors are absorbed predominantly in the proximal gastrointestinal tract and demonstrated bidirectional metabolic interactions between host tissues and the gut microbiome [23]. Circulating host-derived NAM can enter the intestinal lumen and support microbial NAD metabolism, while the gut microbiota can convert NAM to NA, which can subsequently contribute to NAD synthesis in host tissues. The same study showed that oral NR can contribute to host NAD synthesis substantially through microbiome-mediated conversion to NA [23]. These findings indicate that systemic responses to NAD precursor supplementation may depend not only on canonical intracellular pathways but also on precursor interconversion and host–microbiome interactions. Importantly, these mechanistic findings were primarily derived from experimental models and should not be assumed to translate quantitatively to humans.

Maintenance of NAD homeostasis may be particularly relevant to RGC axons because disruption of NAD metabolism is closely linked to axonal degeneration. NMNAT2 contributes to axonal NAD maintenance, and loss of NMNAT2 alters NAD metabolism and promotes activation of the SARM1-dependent axon degeneration pathway [22,24,25]. Activated SARM1 consumes NAD^+^ and initiates a metabolic program leading to rapid NAD depletion and axonal degeneration [22].

Experimental glaucoma provides further support for the relevance of these pathways. Age-related reductions in NAD availability and altered mitochondrial metabolism have been associated with increased RGC susceptibility to glaucomatous stress, whereas restoration of NAD availability through NAM supplementation provides substantial neuroprotection in experimental models [11]. These findings further support the investigation of NAD precursor supplementation as a metabolic strategy in glaucoma, while emphasizing that individual precursors may differ in their absorption, metabolism, tissue distribution, and biological effects.

## 2. Materials and Methods

This systematic review was conducted and reported in accordance with the Preferred Reporting Items for Systematic Reviews and Meta-Analyses (PRISMA) 2020 statement [26]. The review protocol was not prospectively registered in PROSPERO, which is acknowledged as a methodological limitation. No separate publicly accessible review protocol was available before study selection. The review question and eligibility criteria were structured according to the Population, Intervention/Exposure, Comparison, and Outcomes (PICO) framework.

### 2.1. Literature Search Strategy

A systematic literature search was conducted in PubMed, Scopus, and the Cochrane Library from database inception to 22 August 2026. The search was updated during manuscript revision to identify recently published studies and to ensure inclusion of the most current human evidence available at the time of the review.

The search strategy combined controlled vocabulary, where available, and free-text terms related to vitamin B3 and NAD metabolism, including “nicotinamide,” “niacinamide,” “nicotinamide riboside,” “nicotinic acid,” “niacin,” “vitamin B3,” “NAD,” and “nicotinamide adenine dinucleotide,” together with terms related to “glaucoma” and “ocular hypertension.” No restriction by publication year was applied. Searches were restricted to English-language publications. Eligibility was subsequently determined during study screening according to the predefined inclusion and exclusion criteria. Reference lists of eligible studies and relevant reviews were additionally screened to identify potentially eligible studies not retrieved by the electronic search.

The complete database-specific search strategies, including the exact date of the final search, applied filters, and number of records retrieved from each database, are provided in Supplementary Table S1.

### 2.2. Eligibility Criteria

Studies were eligible if they investigated human participants aged ≥18 years with glaucoma or ocular hypertension and evaluated nicotinamide (NAM) supplementation, NAM-containing or NAD-targeted metabolic interventions, or NAM/NAD-related metabolic alterations directly relevant to glaucoma. Given the limited and heterogeneous human literature, both interventional and observational studies were considered eligible, while their findings were evaluated separately according to study design and research question.

Eligible interventional studies included randomized and non-randomized clinical studies evaluating oral NAM, either alone or in combination with another metabolic intervention, as well as other NAD-targeted interventions directly relevant to glaucoma. Eligible observational studies included investigations of circulating or intracellular NAM/NAD-related biomarkers or mitochondrial metabolic parameters directly related to NAD homeostasis in relation to glaucoma status or progression.

Studies were required to report at least one relevant clinical, functional, electrophysiological, metabolic, or safety outcome. Outcomes of interest included visual field parameters, electrophysiological measures of retinal function, glaucoma status or progression, systemic or cellular NAM/NAD-related biomarkers, mitochondrial function, and treatment-related adverse events.

Comparators, where applicable, included placebo, control treatment, untreated or healthy control groups. A comparator was not mandatory for eligible observational studies when the study design otherwise provided relevant human evidence addressing the review question.

Studies evaluating dietary vitamin B3 or niacin intake without direct assessment of NAM supplementation, a NAM/NAD-targeted intervention, or NAM/NAD-related metabolic parameters were excluded from the systematic evidence synthesis. Animal and exclusively in vitro studies were also excluded, although selected experimental studies were considered in the Introduction and Discussion solely to provide biological and mechanistic context. Reviews, systematic reviews, meta-analyses, editorials, commentaries, conference abstracts, case reports, and studies without extractable outcomes relevant to the review question were excluded. Only full-text articles published in peer-reviewed journals in English were eligible. No restriction based on publication year was applied.

Because the review was designed to distinguish evidence regarding therapeutic supplementation from supportive human metabolic evidence, interventional and observational studies were analyzed separately and were not considered equivalent sources of evidence for treatment efficacy. The eligibility criteria are summarized in Table 1.

### 2.3. Study Selection and Data Extraction

After removal of duplicate records, two reviewers independently screened titles and abstracts according to the predefined eligibility criteria. Full-text articles were retrieved for potentially eligible studies and independently assessed for inclusion. When eligibility could not be established from the title and abstract alone, the full-text article was reviewed. Disagreements were resolved by consensus, with involvement of a third reviewer when necessary. Records identified through the updated search conducted on 22 August 2026 were screened using the same eligibility criteria and study-selection procedures.

Data were extracted using a predefined standardized framework. Extracted information included first author, year of publication, country, study design, sample size, participant characteristics, glaucoma subtype, intervention or exposure, NAM or other NAD-related regimen where applicable, comparator, treatment or follow-up duration, outcome measures, principal findings, and reported adverse events.

For randomized and other interventional studies, particular attention was given to NAM dose and administration schedule, concomitant metabolic interventions, visual field outcomes, electrophysiological measures, intraocular pressure, treatment duration, adherence, and safety findings. For observational studies, extracted information included the type of exposure or biomarker investigated, NAM or NAD-related metabolic findings, mitochondrial parameters where applicable, associations with glaucoma prevalence or progression, adjustment for potential confounders, and major methodological limitations.

Because studies evaluating NAM-containing or NAD-targeted supplementation and those investigating NAM/NAD-related biomarkers or mitochondrial metabolic alterations address different clinical questions, their findings were extracted and synthesized separately.

### 2.4. Risk of Bias Assessment

Risk of bias was assessed independently by two reviewers, with disagreements resolved by consensus. The assessment approach was selected according to study design.

Randomized controlled trials were evaluated using the revised Cochrane Risk of Bias tool (RoB 2), which assesses bias arising from the randomization process, deviations from intended interventions, missing outcome data, measurement of the outcome, and selection of the reported result [27]. For randomized crossover trials, the assessment additionally considered methodological issues specific to the crossover design, including potential period and carryover effects.

Non-randomized interventional studies were evaluated using the Risk Of Bias In Non-randomized Studies of Interventions (ROBINS-I) tool [28]. The domains considered were bias due to confounding, selection of participants, classification of interventions, deviations from intended interventions, missing data, measurement of outcomes, and selection of the reported result.

For observational exposure and biomarker studies, risk of bias was assessed using a domain-based approach tailored to the study design. The domains considered were participant selection, exposure or biomarker measurement, outcome assessment, confounding, missing data, and selective reporting. These studies were evaluated as observational evidence and were not interpreted as providing estimates of therapeutic efficacy.

When multiple publications arose from the same underlying study population, related reports were considered together when evaluating potential sources of bias and were not treated as independent studies solely on the basis of separate publication.

Given the small number of eligible studies and the heterogeneity of study designs, risk-of-bias findings were presented at the individual-study level rather than as percentage-based summary plots. This approach was chosen to avoid implying quantitative precision from proportions derived from a very small number of studies.

### 2.5. Data Synthesis

Because of the limited number of eligible interventional studies and substantial heterogeneity in study design, glaucoma subtype, treatment regimen, duration of supplementation, comparator, and outcome definition, a quantitative meta-analysis was not considered methodologically appropriate. In particular, available randomized studies differed in whether NAM was administered alone or in combination with another metabolic intervention, and crossover and parallel-group designs could not be assumed to provide directly interchangeable effect estimates.

Accordingly, results were synthesized narratively, with interventional and observational evidence considered separately. For randomized trials, the direction, magnitude, and statistical significance of reported treatment effects were extracted as presented in the original publications, without recalculation of standardized effect sizes from data that did not adequately account for the underlying study design.

For crossover studies, results were interpreted according to the analyses reported by the original investigators, thereby preserving the paired nature of the data and avoiding treatment of within-participant observations as independent parallel groups.

Observational studies were synthesized according to the type of evidence provided, including circulating NAM concentrations, cellular or systemic mitochondrial/NAD-related metabolic parameters, and associations with glaucoma status or progression.

No statistical imputation was performed for missing or incompletely reported data. Studies with incomplete quantitative reporting were retained in the narrative synthesis when sufficient information was available for meaningful interpretation.

### 2.6. Assessment of Reporting Bias

Because the number of eligible studies was small and the included studies were clinically and methodologically heterogeneous, formal assessment of publication bias using funnel plots or statistical tests for funnel-plot asymmetry was not considered appropriate. The possibility of publication and selective reporting bias was instead considered qualitatively when interpreting the overall certainty of the evidence.

## 3. Results

### 3.1. Study Selection

The updated literature search identified 561 records across PubMed (*n* = 83), Scopus (*n* = 453), and the Cochrane Library (*n* = 25). Following deduplication, 470 records underwent title and abstract screening. Of these, 14 potentially relevant reports were sought for full-text assessment. One recently published report could not be retrieved in full text, leaving 13 reports that were assessed for eligibility.

Following full-text assessment, 13 eligible reports, representing eight unique human studies, were included in the systematic review. Multiple publications arising from the same underlying study population were linked and treated as reports of a single study rather than as independent studies.

The updated search identified additional recently published human evidence that was not available within the time frame of the original search. The final evidence base comprised randomized clinical trials, non-randomized supplementation studies, and observational human studies evaluating NAM-containing or NAD-targeted interventions, NAM/NAD-related biomarkers, and mitochondrial metabolic alterations in relation to glaucoma. Because interventional and observational studies addressed distinct therapeutic and mechanistic questions, their findings were synthesized separately.

**Figure 1:**
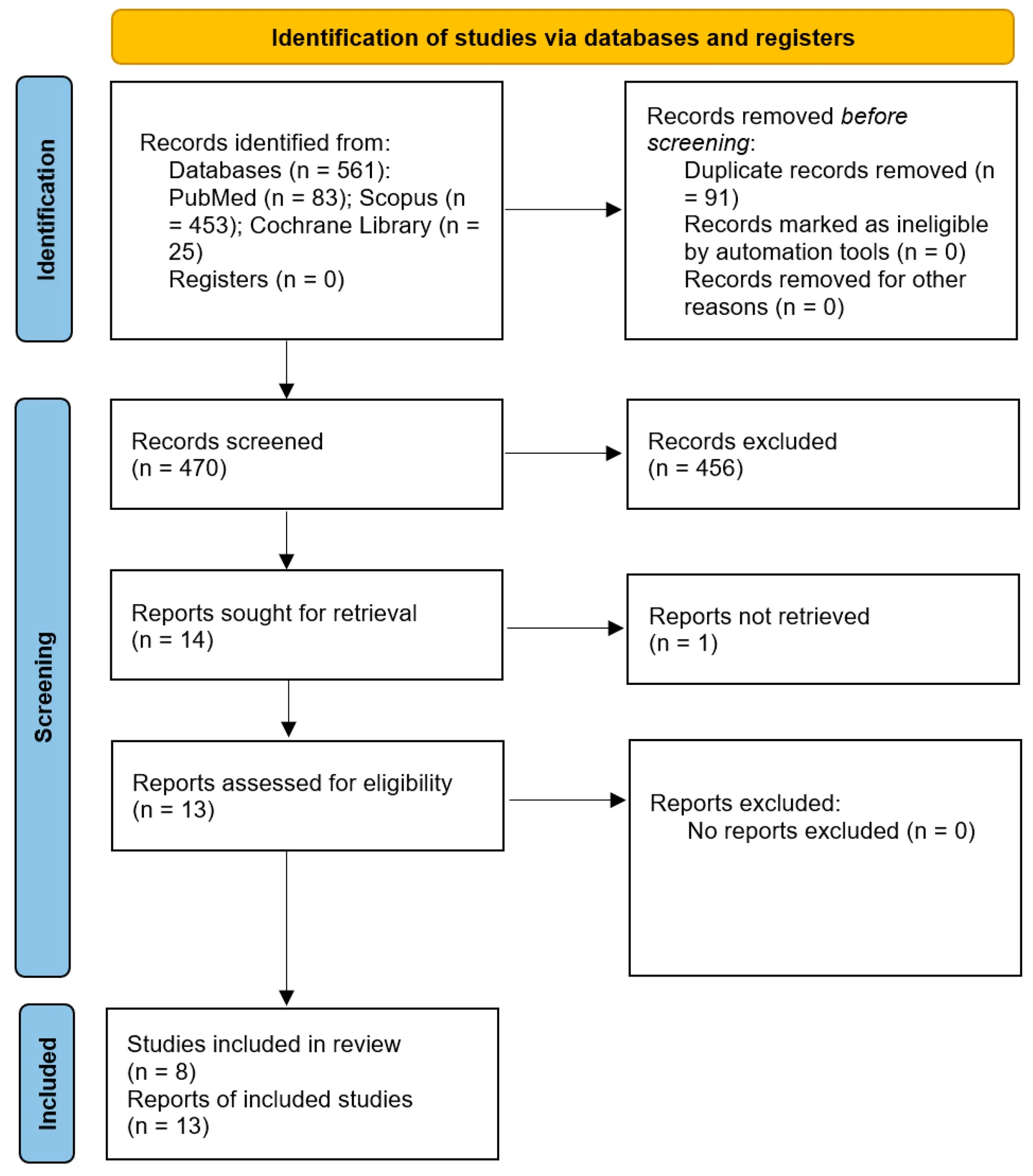
PRISMA 2020 flow diagram illustrating the study selection process.

The complete study-selection process, including duplicate removal, screening, report retrieval, and full-text assessment, is presented in the PRISMA 2020 flow diagram (Figure 1).

### 3.2. Characteristics of Included Studies

The included studies were heterogeneous with respect to study design, glaucoma phenotype, metabolic intervention or exposure, treatment regimen, duration of follow-up, and outcome assessment. The evidence base comprised randomized clinical trials of oral nicotinamide (NAM), prospective and retrospective non-randomized supplementation studies, and observational studies evaluating systemic NAM/NAD-related metabolic and mitochondrial alterations in glaucoma. Multiple publications derived from the same underlying study population were considered as separate reports of a single study rather than as independent studies.

Among the randomized interventional evidence, Hui et al. [29] investigated oral NAM as adjunctive therapy in patients with treated glaucoma using a randomized crossover design. NAM was administered at 1.5 g/day for 6 weeks followed by 3.0 g/day for an additional 6 weeks. NAM supplementation was associated with improvement in inner retinal function, assessed using the photopic negative response (PhNR), together with improvement in selected visual field parameters.

De Moraes et al. [30] evaluated a combined metabolic intervention consisting of oral NAM and pyruvate in patients with treated open-angle glaucoma. Participants received escalating doses of NAM (1000–3000 mg/day) together with pyruvate (1500–3000 mg/day). Compared with placebo, the intervention was associated with a greater number of improving visual field test locations and favorable changes in selected visual field parameters. Because NAM was administered together with pyruvate, the observed effects cannot be attributed specifically to NAM alone. A subsequent post hoc analysis of the same randomized trial further characterized the topographical distribution of visual field improvement and was therefore considered a secondary report rather than an independent study.

The updated search additionally identified the randomized, double-masked, placebo-controlled crossover trial by Ha et al. [31] in 53 patients with normal-tension glaucoma receiving intraocular pressure-lowering treatment. Participants received NAM at 1 g/day for 6 weeks followed by 2 g/day for a further 6 weeks. NAM was associated with significantly greater improvement in PhNR and electroretinographic B-wave responses compared with placebo, whereas no significant between-group differences were observed for global visual field indices, including mean deviation, pattern standard deviation, and visual field index.

Additional non-randomized interventional evidence was identified in the updated search. A prospective study by Gustavsson et al. [32] evaluated short-term high-dose NAM supplementation in patients with different glaucoma subtypes and healthy controls. Participants received NAM at 1.5 g/day for 1 week followed by 3.0 g/day for 1 week. Subsequent publications from the same study population investigated systemic metabolic responses, mitochondrial DNA content, and circulating extracellular nicotinamide phosphoribosyltransferase (eNAMPT) [33,34]; these publications were treated as secondary reports of the same underlying study rather than as independent studies.

Nicola et al. [35] evaluated longer-term niacinamide supplementation at 500 mg/day for 6 months in a prospective, non-randomized, single-arm study of patients with primary open-angle glaucoma. A secondary report from the same study population evaluated structural and electrophysiological outcomes [36]. Because these reports originated from the same clinical study, they were considered jointly when characterizing the evidence.

A retrospective uncontrolled study by Visalli et al. [37] evaluated a combined metabolic strategy involving nicotinamide riboside and berberine in patients with primary open-angle glaucoma receiving stable topical treatment. Given the absence of an untreated control group and the combined nature of the intervention, these findings were considered exploratory and were interpreted separately from randomized evidence.

Among the observational evidence, Kouassi Nzoughet et al. [20] reported significantly lower plasma NAM concentrations in patients with primary open-angle glaucoma compared with matched controls. Related metabolomic analyses provided additional evidence of systemic metabolic alterations associated with glaucoma.

Petriti et al. [21] investigated systemic mitochondrial function in glaucoma and reported impaired peripheral blood mononuclear cell respiratory function in patients with glaucoma, with poorer mitochondrial respiratory performance associated with faster visual field progression. NAD-related analyses provided additional evidence linking systemic bioenergetic dysfunction and altered NAD metabolism with glaucoma progression. These observational findings were considered mechanistic evidence and not evidence of supplementation efficacy.

The characteristics and principal findings of the included studies and their associated reports are summarized in Table 2.

### 3.3. Risk of Bias

**Figure 2:**
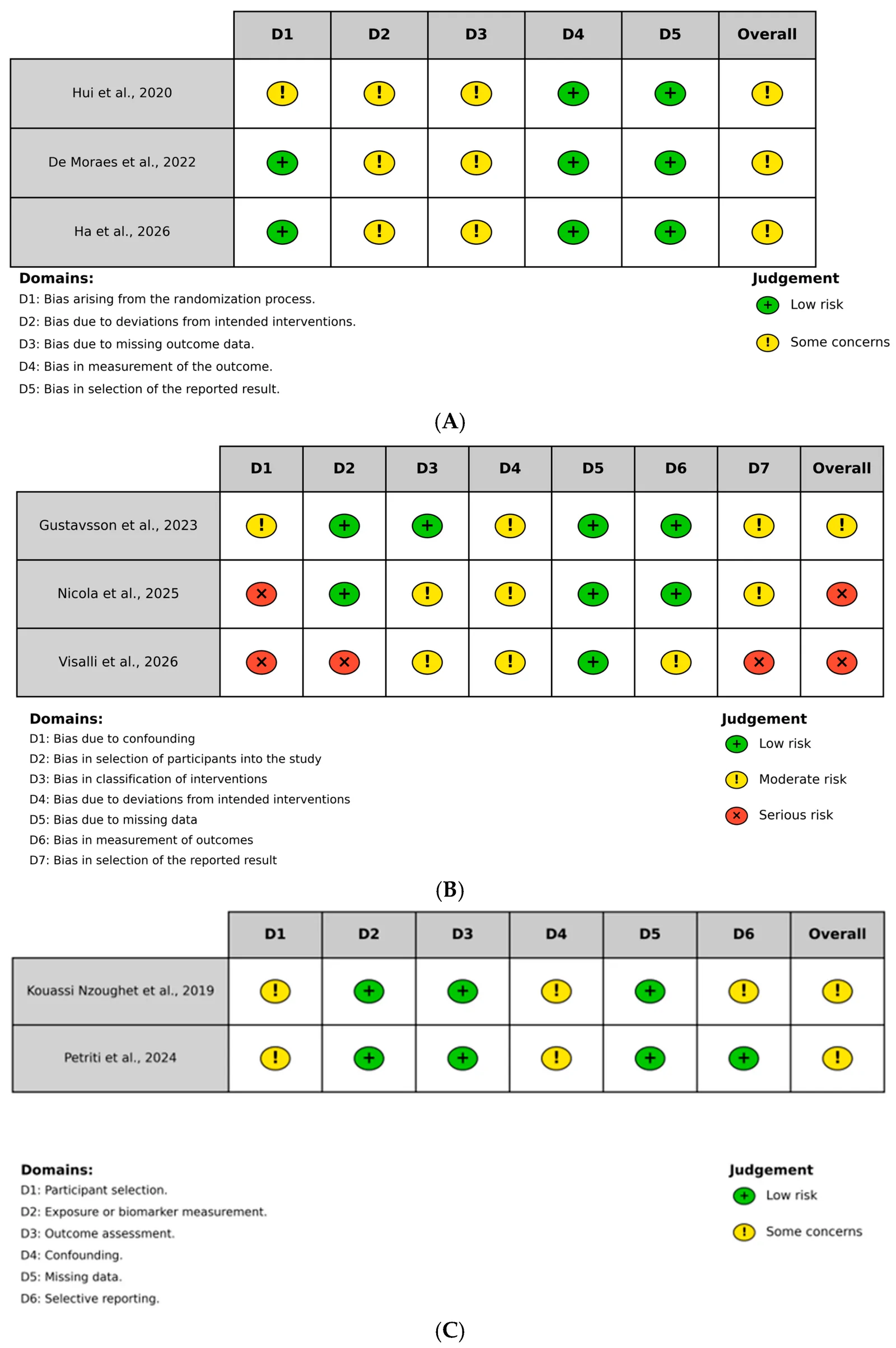
(**A**) Risk-of-bias assessment of randomized controlled trials using the revised Cochrane Risk of Bias tool (RoB 2) [29,30,31]. (**B**) Risk-of-bias assessment of non-randomized interventional studies using ROBINS-I [32,35,37]. (**C**) Risk-of-bias assessment of observational exposure and biomarker studies [20,21].

Risk-of-bias assessments are presented in Figure 2A–C according to study design. Overall, the three randomized trials were judged to raise some concerns, primarily because of incomplete outcome data, analyses restricted to participants completing the relevant study periods, and, for the crossover trials, methodological considerations related to the absence of a washout period. Risk related to outcome measurement and selective reporting was generally considered low, given the use of masked outcome assessment, predefined outcome measures, and prospective trial registration where available.

Among the non-randomized interventional studies, risk of bias ranged from moderate to serious, with the main concerns arising from confounding, participant selection, and the absence of randomized concurrent control groups in some studies. These limitations were particularly relevant when interpreting changes observed after supplementation as treatment effects.

The observational exposure and biomarker studies also showed methodological limitations, predominantly related to residual confounding and participant selection. Biomarker and outcome measurements were generally considered adequately performed, whereas the observational nature of these studies limited causal inference. Risk-of-bias judgments for individual studies are shown in Figure 2C.

### 3.4. Interventional Evidence

Given the clinical and methodological heterogeneity of the available interventional studies, findings were evaluated individually rather than quantitatively pooled.

In the randomized crossover trial by Hui et al. [29], short-term oral NAM supplementation was associated with improvement in electrophysiological measures of inner retinal function and selected visual field parameters. The findings were interpreted according to the analyses reported by the original investigators, preserving the paired nature of the crossover design.

In the phase II randomized clinical trial by De Moraes et al. [30], the combination of NAM and pyruvate was associated with short-term improvement in selected visual field outcomes compared with placebo. However, because NAM and pyruvate were administered concurrently, the specific contribution of NAM cannot be isolated. Furthermore, the short treatment duration limits conclusions regarding sustained neuroprotection or modification of long-term glaucoma progression.

More recently, Ha et al. [31] evaluated oral NAM in 53 patients with normal-tension glaucoma in a double-masked, placebo-controlled crossover randomized clinical trial. NAM was administered for 12 weeks at 1 g/day for the first 6 weeks and 2 g/day for the subsequent 6 weeks. Compared with placebo, NAM produced significantly greater increases in photopic negative response peak-to-trough amplitude and B-wave amplitude (*p* = 0.045 and *p* = 0.032, respectively). In contrast, no significant between-group differences were observed in mean deviation, pattern standard deviation, or visual field index, indicating electrophysiological improvement without a detectable short-term visual field benefit.

Additional non-randomized human studies provided complementary evidence on the effects of NAM supplementation. Gustavsson et al. [32,33,34] evaluated 90 patients with glaucoma and 30 healthy controls undergoing a 2-week accelerated NAM regimen (1.5 g/day during the first week and 3.0 g/day during the second week). Short-term NAM treatment was associated with small increases in retinal vascular parameters measured by OCT angiography, although the authors considered these changes of limited clinical relevance. Subsequent analyses of the same underlying cohort demonstrated marked increases in circulating NAM and related metabolites after supplementation and explored associated changes in systemic mitochondrial and NAD-related biomarkers. These secondary reports were considered mechanistic extensions of the original clinical cohort rather than independent intervention studies.

Longer-term non-randomized evidence was provided by Nicola et al. [35,36], who investigated six months of oral niacinamide supplementation in patients with primary open-angle glaucoma in a prospective single-arm clinical setting. These studies explored patient-reported quality of life as well as structural and electrophysiological outcomes; however, the absence of a randomized concurrent control group limits attribution of longitudinal changes specifically to supplementation.

Finally, Visalli et al. [37] retrospectively evaluated patients with primary open-angle glaucoma receiving combined nicotinamide riboside and berberine supplementation in addition to stable topical hypotensive therapy. Functional stabilization of the visual field and structural preservation of the retinal nerve fiber layer were reported over six months. However, the retrospective design and the absence of groups receiving nicotinamide riboside or berberine alone prevent attribution of the observed findings specifically to NAD-targeted supplementation or determination of a synergistic effect.

Taken together, the interventional evidence suggests that NAM-containing metabolic strategies may produce short-term functional, electrophysiological, vascular, and metabolic changes in glaucoma. Nevertheless, the evidence remains insufficient to establish sustained neuroprotection or modification of disease progression. Differences in glaucoma phenotype, intervention composition, dosing regimen, treatment duration, study design, and outcome definition, together with the limited number of controlled trials and the presence of multiple reports derived from the same study populations, precluded a methodologically robust quantitative synthesis.

### 3.5. Observational and Mechanistic Human Evidence

Observational studies provided complementary evidence regarding NAM/NAD-related metabolism and mitochondrial function in glaucoma but were not interpreted as evidence of treatment efficacy.

Kouassi Nzoughet et al. [20] demonstrated lower circulating NAM concentrations in patients with primary open-angle glaucoma than in control participants, suggesting an association between glaucoma and altered systemic NAM availability.

Petriti et al. [21] reported that impaired peripheral blood mononuclear cell mitochondrial respiratory function was associated with faster visual field deterioration in glaucoma. These findings provide human mechanistic evidence supporting an association between systemic mitochondrial dysfunction, NAD-related metabolism, and glaucomatous disease progression.

Overall, these observational and mechanistic findings support the biological plausibility of altered NAM/NAD metabolism and mitochondrial dysfunction in glaucoma. However, they do not establish a causal relationship or demonstrate the clinical efficacy of NAM supplementation.

## 4. Discussion

This systematic review summarizes the available human evidence on nicotinamide (NAM), NAD-targeted supplementation strategies, and NAD-related metabolic alterations in glaucoma. Overall, the evidence supports a biologically plausible relationship between impaired NAD metabolism, mitochondrial dysfunction, and glaucomatous neurodegeneration and suggests that NAM-containing interventions may produce short-term functional and metabolic effects. However, the clinical evidence remains limited and heterogeneous, and the available findings should be regarded as hypothesis-generating rather than sufficient to support routine NAM supplementation as a neuroprotective treatment for glaucoma.

The interventional evidence derives from randomized and non-randomized studies that differ substantially in glaucoma phenotype, intervention composition, dosing regimen, treatment duration, study design, and outcome assessment. Hui et al. [29] reported that short-term oral NAM supplementation in patients with treated early-to-moderate glaucoma improved inner retinal function, as assessed by the photopic negative response (PhNR), together with selected visual field parameters. De Moraes et al. [30] reported favorable short-term visual field changes following combined NAM and pyruvate supplementation. Importantly, NAM was administered together with pyruvate in that trial, with escalating doses of NAM from 1000 to 3000 mg/day and pyruvate from 1500 to 3000 mg/day over a 3-week treatment period. Consequently, the observed effect cannot be attributed specifically to NAM.

More recently, Ha et al. [31] extended the randomized evidence to patients with normal-tension glaucoma. In this placebo-controlled crossover trial, NAM supplementation was associated with significant improvement in electrophysiological measures of retinal function, including PhNR and B-wave amplitudes, whereas no significant between-group differences were detected in visual field mean deviation, pattern standard deviation, or visual field index. This distinction is clinically relevant because it suggests that short-term electrophysiological changes should not be interpreted as evidence of visual field preservation or disease modification. Collectively, the randomized trials provide preliminary evidence of short-term functional effects but do not establish sustained neuroprotection or slowing of glaucomatous progression.

Non-randomized interventional studies provide complementary but methodologically weaker evidence. Short-term supplementation studies have explored retinal vascular and systemic metabolic responses to NAM, while longer-term investigations have assessed structural, electrophysiological, functional, and patient-reported outcomes. Other NAD-targeted strategies, including nicotinamide riboside-containing supplementation, have also been investigated. Although these studies broaden the range of potentially relevant biological and clinical outcomes, their interpretation is constrained by the absence of randomized concurrent controls in some studies, retrospective or single-arm designs, potential confounding, and, in some cases, combined interventions that prevent attribution of observed effects to an individual component. Moreover, multiple publications derived from the same underlying study populations should be interpreted as complementary analyses rather than independent replication of treatment effects.

The observational evidence provides further support for a relationship between NAM/NAD metabolism, mitochondrial function, and glaucoma but cannot establish therapeutic efficacy. Kouassi Nzoughet et al. [20] reported lower circulating NAM concentrations in patients with primary open-angle glaucoma compared with controls, suggesting altered systemic NAM availability in glaucoma. Petriti et al. [21] further demonstrated an association between impaired peripheral blood mononuclear cell mitochondrial respiratory function, altered NAD-related metabolic status, and faster visual field deterioration. Collectively, these findings suggest that systemic metabolic and mitochondrial alterations may accompany glaucoma and provide mechanistic support for NAD-targeted therapeutic investigation; however, observational associations cannot establish causality or demonstrate that correction of these abnormalities modifies disease progression.

The biological rationale for targeting NAD metabolism is supported by experimental evidence demonstrating a relationship between NAD availability, mitochondrial resilience, and retinal ganglion cell survival. In aged and genetically susceptible mouse models of glaucoma, retinal NAD decline and mitochondrial vulnerability occur early in the disease process, whereas NAM supplementation increases metabolic resilience and provides substantial protection against retinal ganglion cell degeneration [11,38]. Experimental studies have also implicated NAD-dependent axonal degeneration pathways, including NMNAT2 depletion and SARM1 activation, in neuronal and axonal injury [22,24,25]. These findings provide a mechanistic basis for clinical investigation of NAD-targeted strategies; however, the magnitude of neuroprotection observed in experimental models cannot be assumed to translate directly to patients with glaucoma.

NAM should also be distinguished from other forms of vitamin B3 and other NAD precursors. Nicotinic acid, NAM, and nicotinamide riboside enter NAD biosynthesis through distinct metabolic routes [8,9,10]. Although nicotinamide riboside and other NAD-boosting approaches have attracted considerable interest, the most established glaucoma-specific clinical evidence to date concerns NAM, while evidence for alternative NAD precursors remains comparatively limited. Emerging evidence also indicates that orally administered NAD precursors may undergo substantial host–microbiome metabolic interconversion before contributing to systemic NAD synthesis [23]. Consequently, different NAD precursors should not be assumed to have equivalent pharmacokinetic, metabolic, or clinical effects, and findings obtained with one precursor should not automatically be extrapolated to another.

Safety is particularly relevant because any neuroprotective strategy for glaucoma would likely require prolonged administration. The available clinical studies provide only limited reassurance regarding short-term tolerability and are insufficient to establish long-term safety. High-dose NAM has previously been associated in other clinical settings with gastrointestinal adverse effects, thrombocytopenia, abnormalities in liver enzymes, and, less commonly, hepatotoxicity [39,40,41]. In the glaucoma setting, Shukla et al. reported drug-induced liver injury during a neuroprotection clinical trial involving high-dose NAM [42]. Subsequently, the American Glaucoma Society and the American Academy of Ophthalmology issued a joint position statement cautioning against unsupervised high-dose NAM supplementation and emphasizing appropriate clinical monitoring [43]. These safety considerations are especially important when gram-level doses are contemplated over prolonged periods and argue against extrapolating short-term tolerability to chronic routine use.

An important strength of the present review is its specific focus on human evidence while clearly distinguishing randomized clinical trials, non-randomized interventional evidence, and observational metabolic and epidemiological studies. This approach allows therapeutic signals to be considered separately from evidence supporting biological plausibility and avoids interpreting observational associations as evidence of treatment efficacy. The present work also extends beyond visual field outcomes by considering electrophysiological measures, structural and vascular parameters, metabolic biomarkers, mitochondrial function, safety, and methodological quality. This distinguishes it from previous narrative reviews that have provided broader discussions of NAD biology, experimental neuroprotection, and potential therapeutic applications [12,13,14,15]. A recent systematic review specifically evaluated the effect of NAM on visual field mean deviation [16]; the present review adopts a broader human-evidence framework and incorporates recently available clinical and mechanistic studies while formally considering study design and risk of bias.

Several limitations should nevertheless be acknowledged. First, the human evidence base remains small, particularly when restricted to randomized supplementation studies. Second, the available trials include relatively modest sample sizes and generally limited treatment durations, reducing statistical power and preventing conclusions regarding sustained neuroprotection or long-term glaucoma progression. Third, substantial clinical and methodological heterogeneity exists across glaucoma phenotypes, intervention composition, NAM dose, treatment duration, study design, comparator, and outcome measures. In particular, the combined administration of NAM and pyruvate in the De Moraes trial [30] prevents attribution of its observed effects specifically to NAM. Fourth, crossover, parallel-group, single-arm, and retrospective studies cannot be treated as methodologically interchangeable, and several reports arise from the same underlying study populations rather than representing independent clinical evidence. These differences, together with the small number of controlled trials and heterogeneous outcome definitions, precluded a methodologically robust quantitative synthesis; therefore, the evidence was synthesized narratively. Fifth, the non-randomized interventional evidence is particularly susceptible to confounding and selection bias, whereas observational associations involving circulating NAM or mitochondrial function cannot establish that correcting these metabolic alterations modifies glaucoma progression. Finally, the review was not prospectively registered in PROSPERO, which represents an additional methodological limitation.

The current evidence therefore supports continued clinical investigation rather than routine therapeutic adoption. Future randomized trials should be adequately powered and of sufficient duration to evaluate clinically meaningful outcomes, particularly rates of visual field and structural progression rather than predominantly short-term surrogate endpoints. Trials should establish dose–response relationships, determine whether observed benefits are attributable specifically to NAM or to combined metabolic interventions, directly investigate alternative NAD precursors where appropriate, and evaluate whether particular glaucoma phenotypes or metabolic profiles identify patients more likely to respond. Standardized hepatic, hematological, metabolic, and ocular safety monitoring should be incorporated, particularly when high-dose or prolonged supplementation is investigated. Longer-term evidence will ultimately be required to determine whether modulation of NAD metabolism can translate biological plausibility and short-term functional signals into sustained retinal ganglion cell neuroprotection and clinically meaningful modification of glaucoma progression.

## 5. Conclusions

Nicotinamide represents a biologically plausible metabolic strategy for glaucoma neuroprotection, supported by experimental and human mechanistic evidence linking altered NAD metabolism, mitochondrial dysfunction, and retinal ganglion cell vulnerability. Emerging clinical evidence suggests that NAM-containing and NAD-targeted interventions may produce short-term functional, electrophysiological, and metabolic effects, while observational studies further support an association between altered vitamin NAM/NAD metabolism, mitochondrial dysfunction, and glaucoma.

However, the current clinical evidence remains limited by the small number of randomized trials, modest sample sizes, short treatment durations, heterogeneous glaucoma populations, differences in intervention composition and dosing regimens, and the predominance of short-term or surrogate outcomes. Importantly, short-term functional or electrophysiological changes cannot yet be interpreted as evidence of sustained neuroprotection or modification of glaucoma progression. Moreover, findings from combined metabolic interventions, such as NAM plus pyruvate, cannot be attributed specifically to NAM, and evidence from non-randomized studies remains susceptible to confounding and other sources of bias.

Accordingly, the available evidence does not currently support routine high-dose NAM supplementation as part of standard glaucoma management. Larger, adequately powered, long-term randomized controlled trials are required to determine whether modulation of NAD metabolism provides sustained neuroprotective benefit, establish optimal dosing and treatment duration, identify patients most likely to benefit, and define the long-term safety profile of chronic supplementation.

## Figures and Tables

**Table 1:** Eligibility criteria applied during study selection.

Criterion	Inclusion Criteria	Exclusion Criteria
Population	Human participants aged ≥18 years with glaucoma, or populations in which glaucoma status or risk was evaluated in relation to NAM or NAD-related metabolic parameters.	Animal studies; exclusively in vitro studies; pediatric populations.
Intervention/Exposure	Oral NAM alone or combined with another metabolic intervention; other NAM/NAD-targeted interventions directly relevant to glaucoma; circulating or intracellular NAM/NAD-related biomarkers; mitochondrial metabolic parameters directly related to NAD homeostasis.	Dietary vitamin B3 or niacin intake without direct assessment of NAM supplementation, a NAM/NAD-targeted intervention, or NAM/NAD-related metabolic parameters; interventions or exposures unrelated to NAM/NAD metabolism.
Comparator	Placebo; control treatment; untreated controls; healthy controls, where applicable.	No specific comparator required for eligible observational studies.
Outcomes	At least one relevant clinical, functional, electrophysiological, metabolic, epidemiological, or safety outcome, including visual field parameters, electrophysiological measures of retinal function, glaucoma status or progression, systemic or cellular NAM/NAD-related biomarkers, mitochondrial function, or treatment-related adverse events.	Studies without extractable outcomes relevant to the review question.
Study design	Randomized and non-randomized interventional studies; observational human studies.	Reviews; systematic reviews; meta-analyses; editorials; commentaries; conference abstracts; case reports.
Publication characteristics	Full-text articles published in peer-reviewed journals; English language.	Non-English publications; publications without full text; non-peer-reviewed material.
Publication period	From database inception to 22 August 2026.	None based solely on publication year.

Abbreviations: NAM, nicotinamide; NAD, nicotinamide adenine dinucleotide.

**Table 2:** Characteristics of human studies included in the systematic review.

Study	Design/Population	Intervention or Exposure	Follow-Up	Main Findings
Hui et al., 2020 [29]	Randomized crossover trial; 57 patients with treated glaucoma	NAM 1.5 g/day for 6 weeks, then 3.0 g/day for 6 weeks; placebo crossover	12 weeks per treatment sequence	Improved PhNR and selected visual-field outcomes during NAM supplementation.
De Moraes et al., 2022 [30] *	Phase 2 randomized placebo-controlled trial; treated open-angle glaucoma	NAM 1–3 g/day + pyruvate 1.5–3 g/day vs. placebo	Median follow-up ≈ 2.2 months	More improvement in visual-field locations with active treatment; effect cannot be attributed to NAM alone.
Ha et al., 2026 [31]	Randomized double-masked placebo-controlled crossover trial; 53 NTG patients	NAM 1 g/day for 6 weeks, then 2 g/day for 6 weeks; placebo crossover	24-week crossover study	Improved PhNR and ERG B-wave responses; no significant between-group differences in MD, PSD, or VFI.
Kouassi Nzoughet et al., 2019 [20] *	Case–control metabolic study; POAG patients and matched controls	Plasma NAM/NAM-related metabolomic assessment	Cross-sectional	Lower plasma NAM in POAG; related metabolomic analyses supported systemic metabolic alterations.
Petriti et al., 2024 [21]	Observational mechanistic study; glaucoma patients and healthy controls	PBMC mitochondrial respiration and NAD-related metabolic assessment	Observational	Impaired mitochondrial respiratory function was associated with glaucoma and faster visual-field progression.
Gustavsson et al., 2023 [32] *	Prospective controlled supplementation study; 90 glaucoma patients + 30 controls	NAM 1.5 g/day for 1 week, then 3.0 g/day for 1 week	2 weeks	Small retinal vascular changes; secondary reports assessed metabolomic, mtDNA, and eNAMPT outcomes.
Nicola et al., 2025 [35] *	Prospective non-randomized single-arm study; 58 POAG patients	Niacinamide 500 mg/day	6 months	Improved glaucoma-related quality of life; a secondary report evaluated structural and electrophysiological outcomes.
Visalli et al., 2026 [37]	Retrospective uncontrolled clinical study; POAG patients on stable topical therapy	Nicotinamide riboside + berberine	6 months	Exploratory functional and structural findings; causal inference limited by the uncontrolled combined-intervention design.

* Multiple publications arising from the same underlying study population were treated as secondary or related reports and were not counted as independent studies. Abbreviations: NAM, nicotinamide; POAG, primary open-angle glaucoma; NTG, normal-tension glaucoma; PhNR, photopic negative response; ERG, electroretinography; MD, mean deviation; PSD, pattern standard deviation; VFI, visual field index; PBMC, peripheral blood mononuclear cell; mtDNA, mitochondrial DNA; eNAMPT, extracellular nicotinamide phosphoribosyltransferase.

## Data Availability

No new data were created or analyzed in this study. Data sharing is not applicable to this article.

## Supplementary Materials

The following supporting information can be downloaded at: https://www.mdpi.com/article/10.3390/nu18172885/s1, Table S1. Complete electronic literature search strategy.
