# Supplementary material for: Nicotinamide Supplementation in Glaucoma: A Systematic Review of Human Studies
Source: Nutrients. 2026 Sep 3;18(17):2885. doi: 10.3390/nu18172885 (PMC13567524; doi:10.3390/nu18172885)
Supplement: Supplementary file 1 [file nutrients-18-02885-s001.zip › nutrients-4468329-supplementary.pdf]

Supplementary Material

Nicotinamide Supplementation in Glaucoma: A Systematic Review of Human Studies

Table S1. Complete electronic literature search strategy.

Complete database-specific search strategies used to identify eligible human studies investigating nicotinamide, vitamin B3, and NAD-related metabolic strategies in glaucoma and ocular hypertension. Searches were conducted from database inception to 22 August 2026. No publication-year restriction was applied. Database-specific language and human-study filters were applied where available.

Database	Complete search strategy	Filters/limits	Date last searched	Records retrieved (n)
PubMed	("Glaucoma"[MeSH Terms] OR glaucoma[Title/Abstract] OR "ocular hypertension"[Title/Abstract]) AND (nicotinamide[Title/Abstract] OR niacinamide[Title/Abstract] OR "nicotinamide riboside"[Title/Abstract] OR "nicotinic acid"[Title/Abstract] OR niacin[Title/Abstract] OR "vitamin B3"[Title/Abstract] OR NAD[Title/Abstract] OR "NAD+"[Title/Abstract] OR "nicotinamide adenine dinucleotide"[Title/Abstract])	English; Humans	22 August 2026	83
Scopus	TITLE-ABS-KEY ("nicotinamide" OR "niacinamide" OR "nicotinamide riboside" OR "nicotinic acid" OR "niacin" OR "vitamin B3" OR "NAD" OR "NAD+" OR "nicotinamide adenine dinucleotide") AND TITLE-ABS-KEY ("glaucoma" OR "ocular hypertension")	English; no publication-year restriction	22 August 2026	453
Cochrane Library	#1 nicotinamide OR niacinamide OR "nicotinamide riboside" OR "nicotinic acid" OR niacin OR "vitamin B3" OR NAD OR "NAD+" OR "nicotinamide adenine dinucleotide"<br>#2 glaucoma OR "ocular hypertension"<br>#3 #1 AND #2	No publication-year restriction	22 August 2026	25

Total records retrieved before deduplication: n = 561.

Abbreviations: NAD, nicotinamide adenine dinucleotide.
